# Cognitive requirements of competing neuro-behavioral decision systems: some implications of temporal horizon for managerial behavior in organizations

**DOI:** 10.3389/fnhum.2014.00184

**Published:** 2014-04-01

**Authors:** Gordon R. Foxall

**Affiliations:** Cardiff Business School, Cardiff UniversityCardiff, UK

**Keywords:** organizational management, decision-making, neuro-behavioral decisions systems, cognitive style, adaption-innovation, picoeconomics

## Abstract

Interpretation of managerial activity in terms of neuroscience is typically concerned with extreme behaviors such as corporate fraud or reckless investment (Peterson, 2007; Wargo et al., 2010a). This paper is concerned to map out the neurophysiological and cognitive mechanisms at work across the spectrum of managerial behaviors encountered in more day-to-day contexts. It proposes that the *competing neuro-behavioral decisions systems (CNBDS) hypothesis* (Bickel et al., 2012b) captures well the range of managerial behaviors that can be characterized as hyper- or hypo-activity in either the limbically-based impulsive system or the frontal-cortically based executive system with the corresponding level of activity encountered in the alternative brain region. This pattern of neurophysiological responding also features in the Somatic Marker Hypothesis (Damasio, 1994) and in Reinforcement Sensitivity Theory (RST; Gray and McNaughton, 2000; McNaughton and Corr, 2004), which usefully extend the thesis, for example in the direction of personality. In discussing these theories, the paper has three purposes: to clarify the role of cognitive explanation in neuro-behavioral decision theory, to propose picoeconomics (Ainslie, 1992) as the cognitive component of competing neuro-behavioral decision systems theory and to suggest solutions to the problems of imbalanced neurophysiological activity in managerial behavior. The first is accomplished through discussion of the role of picoeconomics in neuro-behavioral decision theory; the second, by consideration of adaptive-innovative cognitive styles (Kirton, 2003) in the construction of managerial teams, a theme that can now be investigated by a dedicated research program that incorporates psychometric analysis of personality types and cognitive styles involved in managerial decision-making and the underlying neurophysiological bases of such decision-making.

## Introduction

Organizational dysfunction has numerous outcomes, from the lack of an appropriate fit between the organization and its environment, through the inappropriate composition of task-based management teams, to the incompatible predispositions and behavioral styles of individual managers. This paper is concerned with the neurophysiological underpinnings of managerial behaviors, in particular with the implications these have for the styles of decision-making and problem-solving managers adopt and their appropriateness for the tasks in hand. Although the neurophysiological basis of behavior in organizations has attracted considerable research attention of late (e.g., Butler and Senior, 2007a, b; Lee et al., 2007; Lee and Chamberlain, 2007), there has been some tendency to address particular aspects of managerial behavior such as trust, cooperation and conflict, reward processing and social interaction rather than to seek a broader framework of conceptualization and analysis for this central aspect of organizational functioning. Worthy as these themes are, this paper proposes that the competing neuro-behavioral decision systems hypothesis (Bickel and Yi, 2008) captures the neurological bases of forms of managerial excess that engender a pathological tendency to avoid risk on one hand and a more reckless tendency to discount the future consequences of current actions on the other.

Theories of managerial behavior and, in particular, prescriptions that derive from them, require a cognitive understanding of the nature of decision-making. The competing neuro-behavioral decisions systems (CNBDS), in common with other neurophysiological accounts of behavior, tend not to have a well-developed cognitive level of exposition. The paper, therefore, examines picoeconomics (Ainslie, 1992), which is similarly couched in terms of temporal discounting, as a candidate for the cognitive component of neuro-behavioral decision theory. Although there is a strong fit, however, picoeconomics provides prescriptions for dealing with the excesses of managerial behavior which befit clinical interventions but are not easily implementable in the context of organizational functioning. In order to overcome this problem, two complementary areas of cognitive-behavioral interaction are examined with a view to increasing understanding of the cognitive component of behavior and suggesting managerial prescriptions, especially for team-building. These are RST (Corr, 2008a) and Adaption-Innovation Theory of cognitive style (Kirton, 1976, 2003), both of which rest on neurophysiological bases that overlap with those on which neuro-behavioral decision theory rests and contribute to the cognitive articulation of the CNBDS hypothesis and the suggestion of meliorating action.

Section Management, Decisions and Cognition discusses the different kinds of management decision and relates them to their possible underlying neurophysiological bases. It also raises the need for clarification of the cognitive dimension of existing theories of neuro-behavioral decision systems and the necessity for managerial application. Section Competing Decision Systems describes the CNBDS hypothesis in detail and relates it to RST and the relevance to managerial decision-making of managers’ temporal horizons. Section The Cognitive Dimension introduces in detail the necessity of a cognitive component of the CNBDS hypothesis and the philosophical implications of speaking of cognition. It lays out criteria for a suitable cognitive component including the necessity of a cognitive theory that proceeds at the personal level of exposition, an intentional account, and potential integration with the economic bases of CNBDS theory, and a close relationship to the basic disciplines in terms of which the theory is couched. Section The Cognitive Dimension also proposes picoeconomics (Ainslie, 1992) as a suitable basis for the cognitive component of neuro-behavioral decision theory and evaluates it in terms of these criteria.

The question of appropriate prescriptions for organizational management is raised in Section Organization-Level Strategies for Changing Managerial Behavior. Although picoeconomics provides insight into the nature of dysfunctional decision-making, its prescriptions are couched in clinical terms and are directed towards the amelioration of addictive behavior. The paper turns, therefore, to the conceptualization of managerial behavior in terms of adaptive-innovative cognitive style (Kirton, 2003) which has broadly similar neurophysiological foundations but which comes equipped with clearer implications for organizational team-building and management. The theory also has implications which are discussed for the understanding of commonplace terms such as strategy, innovation and structure. Overall, the integration of neuro-behavioral decision systems with picoeconomics, RST and adaptive-innovative cognitive style suggests a theory of managerial behavior in organizations which comprehends and proposes means of overcoming problems of dysfunction due to inappropriate temporal horizons (Foxall, 2010).

## Management, decisions and cognition

### Kinds of management decision

Some managerial behaviors patently fail to achieve the goals of the organization in which they are performed, leading often to the downfall of the managers who are responsible for them and sometimes to the failure of the entire organization in which the arise. The hasty shredding of documents of forensic significance, for instance, which has recently figured in more than one dramatic wind-up of a corporation is maladaptive not only for the stakeholders but for the firm itself as a continuing legal entity. For the managers employed by the organization, whether or not they were involved in the termination with extreme prejudice of the documents involved, the maladaptive actions of a few may mean at the very least the interruption of careers. The apparent greed and excessive seeking of immediate reward that accompanied and partially caused the financial crisis of 2008 provides another graphic illustration of the catastrophic effects of maladaptive managerial behavior (Wargo et al., 2010a). This extreme form of maladaptive managerial behavior illustrates vividly the immediacy that motivates some actions within organizations (Peterson, 2007). The informed planning of long-term business operations, in the absence of intrusions caused by short-term concerns, and the timely implementation of strategic intentions, represent the opposite extreme.

It is most probable that neither of these scenarios will figure in the careers of most managers but temporal horizons nevertheless are the hallmark of most managerial activities. Some are most accurately characterized as impulsive; others as planned. This categorization does not correspond exactly to the idea of functional decisions on the one hand, those that meet the goals of the organization and its members, versus dysfunction decisions on the other, those that have outcomes that are contrary to such goals. But it seems reasonable to argue that the majority of impulsive decisions have some dysfunctional consequences, while the majority of planned decisions are functional in the sense defined. It is not helpful to write off the dysfunctional behavior as simply “irrational”: it has its own logic and we should seek its causes just as we seek those of its antithesis. A unified neuroscientific framework within which to pursue these ends is required. First, however, it is necessary to define more closely the range of decisions with which we are concerned.

Two classifications of decisions have proved remarkably resilient and are particularly relevant to the present discussion because they are closely related to temporal horizons: administrative, operating and strategic decisions (Ansoff, 1965) and Simon’s (1965) distinction between programmed and non-programmed decisions. Administrative decisions tend to be routine and to have short time frames. Strategic decisions are, by contrast, long-term and concern the product-market scope of the enterprise, which involves such considerations as diversification policy, the definition of the business, the nature of customer behavior now and in the future, and the integration of the key business areas, namely marketing and innovation (Drucker, 2007). Operating decisions are derived from strategic decisions that have been taken and entail the implementation not only of current interfaces with the business environment, such as the management of marketing mixes, but also the implementation and management of appropriate administrative practices. Programmed decisions are those that are sufficiently routine to have attracted tried and tested, rule-of-thumb decisions systems; so predictable and delegable are these matters that some authorities question whether they entail decision-making at all. Non-programmed decisions are those that arise de novo in the wake of required responses to unstructured situations: new governmental regulations, novel market requirements, radical changes in a competitor’s behavior, and so on. These are generally top-management responsibilities.

Although it is true that most administrative decisions are well-programmed, most strategic decisions non-programmed, and operating decisions a mixture of the two, there are programmed and non-programmed aspects of all three types of decision identified by Ansoff. The key question is what level of management is likely to be involved in each decision type. By and large, administrative decisions can be delegated and taken therefore by relatively junior managers. The repetitiveness that characterizes them suggests that they entail a limited temporal purview which recurs each time they are taken. Indeed, given the extent to which they can be programmed, it is arguable that they are not decisions at all. Strategic decisions are almost by definition unprogrammable and are the domain of senior managers responsible for the overall policy, strategic scope and strategic direction of the enterprise. These decisions, which entail very long-term perspectives on how the firm will develop are almost by definition made in a context of uncertainty. They of course have implications for the administrative and operating decisions that flow from them. Operating decisions are typically the province of middle managers. Although they refer to a time period when relatively accurate assumptions can be made about the product and factor markets in which the firm operates, they are subject to unpredictable fluctuations, e.g., in the behavior of competitors, which necessitate one-off tactical decisions. The temporal horizons of such decisions may vary from the immediate future to short market cycles.

Another way of looking at these decisions is that administrative and to a large extent operating decisions have a pre-existing framework of conceptualization and analysis within which they can be resolved as they arise; in the case of genuinely strategic decisions, it is necessary to construct such a framework coterminously with the initial decision process. It also has to be recognized that once strategic decisions have been made and a suitable decision framework established, the managerial work involved in such decisions takes on an increasingly routine aspect. It is a myth to think that strategic decision-making involves a root and branch analysis of opportunities and capabilities with each planning cycle: many strategic decisions are made recurrently with only small changes in managerial outlook involved on each occasion. This is of course, given the changing market, technological and competitive environments that are the context of such decisions, a source of danger if the firm fails to monitor its strategic space.

From the point of view of the organization, the overall object with respect to decision-making will be to reach an acceptable balance among administrative, operating, and strategic decision-making so that each kind of decision is made in a timely manner and coordinated with the taking of the other kinds of decision. This state of affairs will ensure that conflict between short-term and long-term organizational goals is minimized. Most analyses of managerial decision-making take this purview. But the social cognitive neuroscience approach to organizational behavior makes it possible to discuss the tensions arising within individual managers’ behavior patterns that makes them more or less suited to undertake the decision tasks we have identified. This does not of course mean anything so simplistic as that there are some managers who are predisposed by their limbic systems to make programmed decisions while others have a propensity to make strategic decisions because of their advanced executive functions (EFs). But what the explanation of managerial behavior in terms of the CNBDS hypothesis (Bickel et al., 2012b) has in common with work on extreme behaviors like addiction, etc., is a willingness to embrace the idea that managers’ activities reflect the degree of balance shown by their impulsive and executive systems especially when hypoactivity of the latter permits hyperactivity of the former. It is to this hypothesis that we now turn.

### Competing neuro-behavioral decision systems

The neuroscientific and especially the neuroeconomic account of managerial behavior in organizations has often concentrated on such matters as trust (Zak, 2004, 2007; Zak and Nadler, 2010), cooperation and conflict (Levine, 2007; Tabibnia and Lieberman, 2007), reward processing (Wargo et al., 2010b); and social interaction (Caldú and Dreher, 2007). All of these have some bearing on the kinds of functional and dysfunctional behavior with which we are concerned.

However, this paper seeks an additional explanation for these behaviors in the competing impulsive and executive decision systems associated with the operations of separate, though related, brain regions in the context of corporate problem solving. These neural areas are also associated with differences of temporal horizon, emotional response to circumstances and the cognitive control of behavior. Much of the work inspired by the CNBDS hypothesis involves addictive behavior, influenced by activity located at the impulsive end of the neural spectrum, in contrast to the more calculated behavior that is associated with the EFs, located towards the other pole, which manifest in planning, foresight and evaluation (Bickel et al., 2006, 2012b). Each neural decision system generates its own rewards, relatively immediate and strongly-emotional in the case of the impulsive system, relatively long-term, considered and cognitive in the case of the executive system (Moll and Grafman, 2011). Could it be that the explanation of maladaptive and adaptive organizational decision-making is to be found in the operation of these systems too?

The suspicion that CNBDS might be implicated in managers’ maladaptive behaviors is especially significant in the case of small entrepreneurial businesses which rely largely on the endeavors of a single prime-mover. That person’s tendency towards either impulsiveness or self-control is likely to be a dominant influence on the effectiveness of the enterprise. A tendency towards impulsiveness is likely to manifest in unplanned responses to momentarily appearing opportunities which are implemented without consideration of the long-term consequences for the firm. Unless such instant reactions are constrained by the exercise of EFs which engender planning, foresight, weighing of the relevant consequences, the balance required to build and maintain a successful organization is unlikely to be forthcoming. Conversely, an exaggerated emphasis on strategic thinking and planning which does not express itself in action to launch business ventures will stymie enterprise. The possibility of imbalance arises in a different manner in the large-scale organization. Large firms face similar imperatives requiring the coordination of strategic planning and operational decision-making but the coordination is considerably more complex since different managers are responsible for these tasks. Complications arise because managers charged with making administrative and operational decisions may show cognitive and managerial styles that are incompatible with those of managers charged with strategic planning.

The clearest operational measure of balance/imbalance between the neural systems is the extent of temporal discounting apparent in the manager’s behavior (Bickel and Yi, 2008; see also Baumesiter and Tierney, 2011). The organization-level goal of achieving and maintaining balance among administrative decisions which are predominantly programmed in Simon’s sense, strategic decisions which are relatively unprogrammed, and operating decisions which are predominantly programmed, but sometimes contain unprogrammed elements, has to be accomplished through managers who are typically responsible for a single kind of decision but who bring a particular personal time horizon to it. While the avoidance of conflict between short-term and long-term objectives is an organizational goal, it is not necessarily within the competence or interests of individual managers.

### The need for conceptual clarification

The CNBDS hypothesis *per se* has not previously been applied to managerial concerns. However, the distinction it makes between the functioning of an impulsive system based on the limbic and paralimbic systems and an executive system based on the prefrontal cortex (PFC), together with the possibility that an imbalance between the operations of the two systems may lead to dysfunctional behavior, is strongly represented in the emerging literature of the neuroeconomics of organizations (e.g., Senior and Butler, 2007a,b; Stanton et al., 2010). What we may refer to as *neuro-behavioral decision theory*, which includes the CNBDS hypothesis, other models such as the somatic marker hypothesis (Damasio, 1994), and the application of similar thinking in management (e.g., Wargo et al., 2010a), appears to be emerging as a research paradigm within which to understand dysfunctional behavior (Klein and D’Esposito, 2007; Michl and Taing, 2010).

The first purpose of this paper is to examine and suggest a solution to a conceptual problem that arises in these analyses, a solution which may have a bearing on the kinds of problem of dysfunctional management mentioned above. Like the CNBDS model itself, the discussion of competing neural systems in the context of organizational management tends to conflate events taking place at the neurophysiological level with the cognitive processes ascribed in order to explain and interpret behavior. We are often assured, for instance, that this part of the brain “evaluates”, “plans”, or “decides”. These terms all describe cognitive operations that belong at a level of exposition that refers to the person as a whole rather than the sub-personal level of neurobiology. Each level is properly described in its own language that obeys particular rules and which points to a separate kind of explanation. To draw this distinction between levels of exposition is not to make ontological distinctions or to invite a dualistic approach: it is simply to make clear that we must speak in quite different ways of the rate of firing of neurons from those we employ in speaking of the way in which a consumer evaluates alternative brands. The argument is that while ontologically we have nothing to work with but material events, in accounting for behavior we need to maintain the distinction between what is happening at the sub-personal level of exposition and how we account for behavior at the personal level.

Part of the difficulty arises from a failure to delineate a cognitive component of neuro-behavioral decision theory and to show how it is related to the sub-personal level of neurophysiological events and the super-personal level of behavioral reinforcement. This paper proposes that Picoeconomics (Ainslie, 1992), which analyses the interaction of motivational states that refer to competing temporal horizons, provides the necessary cognitive level of exposition. If this incorporation of picoeconomics as a cognitive level of exposition for neuro-behavioral decision theory is successful, it suggests a means of overcoming problems of dysfunctional managerial behavior that are due to hyperactivity of the impulsive system aided and abetted by hypoactivity of the executive system.

### The need for managerial application

The kind of extreme decision-making involved in corporate fraud or the reckless investing that brings whole economic systems low is comparatively rare. In any case, while neurophysiological processes can explain the behavior of individual participants in such dramas, the opportunity so to act and the far-reaching consequences of such decision-making are likely to be determined by structural factors and special events that lie perhaps beyond the immediate purview, and certainly beyond the control, of the decision-makers themselves (Bailey, 2007; Yeats and Yeats, 2007). An important focus of this paper is on understanding better the nature of decision-making by managers who are, by comparison, involved in more day-to-day corporate management.

The decisions that managers are required to make vary in terms of the cognitive level they demand, including level of intelligence and capacity to cope with complexity. They differ also in terms of their paradigmatic context: at the extremes, some decisions are solvable within the framework of assumption, behavioral norms and market structure that has prevailed hitherto while others require that assumption, behaviors, structures and other variables be reconceptualized and perhaps even re-created. We cannot take all of these factors into consideration but we can speak in terms of the decision styles of managers which have a bearing on their likelihood of success in tackling the various kinds of decision with which they are confronted. Our task is to understand better the causal fabric of the environment within which managers operate (the “super-personal level of exposition”) and the influence of neurophysiology on their behaviors (the “sub-personal level”).

The paper next examines the CNBDS thesis in greater depth, relating it as appropriate to managerial behavior and concerns (Section Competing Decision Systems). This is a prelude to its discussing the cognitive requirements of the model and evaluate picoeconomics as its cognitive component (Section The Cognitive Dimension). Once that is achieved, it is possible to consider the application of the insights of picoeconomics and adaption—innovation theory in addressing problems of managerial dysfunction and the research agenda that emerge from these approaches (Section Organization-Level Strategies for Changing Managerial Behavior).

## Competing decision systems

### Overview of the hypothesis

The CNBDS hypothesis rests on the somewhat simplifying assumption that a “limbic system” can be coherently identified which is differentially implicated in emotional responding and that a cortical area, differentially implicated in judgment, planning and other cognitive activities, can also be identified. Although the reality is undoubtedly more complicated than this—neural activations are seldom exclusive to one part of the brain—the dichotomy is retained here for ease of exposition with regard to the CNBDS hypothesis and for the sake of continuity with a wider literature (cf. however Lawrence and Calder (2004) with Ross (2012)). Bickel’s hypothesis suggests that the degree of addictiveness exhibited in behavior reflects the balance of activity in these two broadly defined brain regions, the first of which, based on the amygdala and ventral striatum, involves the distribution of dopamine (DA) during reinforcement learning, while the second, residing in the PFC, is implicated in the evaluation of rewards and their outcomes (Walton et al., 2011; see also Dayan, 2012; Symmonds and Dolan, 2012).

The *impulsive system* inheres in the amygdala and ventral striatum, a midbrain region concerned with the valence of immediate results of action, and is liable to become hyperactive as a result of “exaggerated processing of the incentive value of substance-related cues” (Bechara, 2005, p. 1459; see also Delgado and Tricomi, 2011). Drug-induced behaviors correlate with enhanced response in this region when the amygdala displays increased sensitization to reward (London et al., 2000; Bickel and Yi, 2008). The *executive system*, located in the PFC is normally associated with planning and foresight but is hypothesized to become hypoactive in the event of addiction; the absence of its moderating function is responsible for the exacerbation of the effects of the hyperactive dopaminergic reward pathway; this imbalance is then viewed as the cause of dysfunctional behavior (Bickel et al., 2011b, 2013). In summary, the CNBDS hypothesis posits that drug seeking results from “amplified incentive value bestowed on drugs and drug-related cues (via reward processing by the amygdala) and impaired ability to inhibit behavior (due to frontal cortical dysfunction)” (Bickel and Yi, 2010, p. 2; see also Jentsch and Taylor, 1999; Rolls, 2009).

### The impulsive system

Before considering the CNBDS hypothesis, it is useful to note Damasio’s (1994) *somatic marker hypothesis* which bases a model of decision-making systems on similar neurophysiological foundations but emphasizes the role of emotion and feelings, downplaying economic considerations. Decision-making reflects the marker signals laid down in bioregulatory systems by conscious and non-conscious emotion and feeling; hence, Bechara and Damasio (2005; see also Bechara et al., 2000) argue that in dealing with decision-making economic theory ignores emotion. Economics is exclusively concerned with “rational Bayesian maximization of expected utility, as if humans were equipped with unlimited knowledge, time, and information processing power”. They point, by contrast, to neural evidence which shows that “sound and rational” decision-making requires antecedent accurate emotional processing (Bechara and Damasio, 2005, p. 336; see also Phelps and Sokol-Hessner, 2012).

Damasio’s (1994) hypothesis is the outcome of brain lesion studies in which damage to the ventromedial prefrontal cortex (vmPFC) was found to be associated with behaving in ways that were personally harmful, especially insofar as they contributed to injury to the social and financial status of the individual and to their social relationships. Although many aspects of these patients’ intellectual functioning such as long-term memory were unimpaired, they were notably disadvantaged with respect to learning from experience and responding appropriately to emotional situations. Moreover, their general emotional level was described as “flat”. Damasio’s observation on these findings was that “the primary dysfunction of patients with vmPFC damage was an inability to use emotions in decision making, particularly decision making in the personal, financial and moral realms” (Naqvi et al., 2006, p. 261). Thus was born the central assumption of the somatic marker hypothesis that “emotions play a role in guiding decisions, especially in situations in which the outcomes of one’s choices, in terms of reward and punishment, are uncertain” (ib.; see also Bechara, 2011). Of relevance here is the finding that the vmPFC may be implicated in activity of the parasympathetic nervous system (PNS), which in contrast to the sympathetic nervous system (SNS) is involved in the explorative monitoring of the environment and the discovery of novelty (Eisenberger and Cole, 2012). This is corroborative of both Damasio’s view and the nature and behavior of the innovative manager discussed below.

Inherent in the somatic marker hypothesis is the attempt to describe not only the separate functions of the brain regions involved in emotional processing but also the interconnections between them (Haber, 2009). The starting point is operant behavior, particularly the mechanisms of reinforcement learning (Daw, 2013; Daw and Tobler, 2013). Specific behaviors eventuate in rewards as a result of which the amygdala triggers emotional/bodily states. These states are then associated via a learning process to the behaviors that brought them about by means of mental representations. As each behavioral alternative is subsequently deliberated upon in the course of decision-making, the somatic state corresponding to it is re-enacted by the vmPFC. After being brought to mind in the course of decision-making the somatic states are represented in the brain by sensory processes in two ways. First, emotional states are related to cortical activation (e.g., insular cortex) in the form of *conscious* “gut feelings” of desire or aversion that are mentally attributed to the behavioral options as they are considered. Secondly, there is an u*nconscious* mapping of the somatic states at the subcortical level—e.g., in the mesolimbic dopaminergic system; in this case, individuals choose the more beneficial option without knowingly feeling the desire for it or the aversiveness of a less beneficial alternative (Ross et al., 2008; see also Di Chiara, 2002; Robbins and Everitt, 2002; Tobler and Kobayashi, 2009).

The rapidity with which the impulsive system acts in propelling behavior is underlined by Rolls’s (2005) theory of emotion in which the reinforcing stimuli consequent on a behavioral act as conditioned stimuli that elicit emotion feelings. The automaticity of this interaction of operant and Pavlovian conditioning may account for behavior in two ways. The emotion feeling may function as an internal discriminative stimulus to increase the probability of the behavior that produced it being reprised; it is equally likely that the emotion feeling is the ultimate reward of the behavior in question and that, by definition, it performs a reinforcing role (Foxall, 2011). Either way, the effects of basic emotions on subsequent responding is immediate and uninfluenced by reflection at the cognitive level. While the criticism of economics shown by the authors of the somatic marker hypothesis appears to rule an economic orientation out of their purview, the CNBDS approach actively builds on insights from operant behavioral economics (Bickel et al., 1999, 2010, 2011a,b; Bickel and Vuchinich, 2000; Bickel and Marsch, 2001; Bickel and Johnson, 2003).

While the somatic marker hypothesis relied in its inaugural stages on lesion studies, the central research technique of cognitive neuropsychology, the work of Rolls (2005) offers confirmation of the role of operant behavior in the emerging paradigm. Recording single neurons’ activity levels, Rolls (2005, 2008) reports that vmPFC neurons respond to the receipt of primary reinforcers such as pleasant-tasting foods. The integrity of the conditioning paradigm is evinced by the finding that devaluation of the reinforcer, for example through satiety, reduced the responses of such areas to these primary reinforcers. fMRI studies also offer corroboration. Gottfried et al. (2003) report that when a predicted primary reinforcer is devalued then vmPFC activity engendered by that reinforcer is reduced. Hence, the vmPFC contributes to the prediction of the reward values of alternative behaviors by reference to their capacity to generate rewarding consequences in prior occasions. Schoenbaum et al. (2003) used lesion and physiological studies to show that this capacity to encode *predictive reward value* depends on an intact amygdala.

The CNBDS model differs in emphasis from Damasio’s somatic marker hypothesis. Their underlying similarity inheres in an acknowledgement that separate functions are performed within the overall impulsive-executive system. But Bickel draws attention to the interconnected operations of the impulsive system and the executive system in the production of behavior (Bickel et al., 2007). The CNBDS hypothesis is open, moreover, to the incorporation of economic analysis in the form of behavioral economics and neuroeconomics (Bickel et al., 2011a). Impulsive action, defined as the choice of a smaller but sooner reward (SSR) over a larger but later reward (LLR), is certainly associated with the over- activation of the older limbic and paralimbic areas, while the valuation and planning of future events and outcomes engages the relatively new (in evolutionary terms) PFC. However, it is the interaction of these areas, which are densely inter-meshed, that generates overt behaviors. The CNBDS hypothesis thus stresses the continuity of the components of the neurophysiologically-based decision system and Bickel’s conception is therefore one of a continuum on which the impulsive and executive systems are arrayed theoretically as polar opponents (Porcelli and Delgado, 2009).

Specifically, Bickel et al. (2012a) identify, in addition to trait impulsivity, four kinds of state impulsivity: behavioral disinhibition, attentional deficit impulsivity, reflection impulsivity and impulsive choice. Trait impulsivity is associated with mesolimbic OFC and correlates with medial PFC, pregenual anterior cingulate cortex (ACC) and ventrolateral PFC; venturesomeness (sensation-seeking) correlates with right lateral orbitofrontal cortex, subgenual anterior cingualate cortex, and left caudate nucleus activations. The concept of trait impulsivity recognizes behavioral regularities that are cross-situationally resilient. Within this broad construct, sensation-seeking or venturesomeness is widely known to be related to a need to reach an optimum stimulation level. Bickel et al. (2012a) associate it with sensitivity to reinforcement, the theory of which has been extensively developed by Corr (2008b) and is discussed in greater detail below. Of the four state impulsivities discussed by Bickel et al. (2012a), behavioral disinhibition is associated with deficiencies in the anterior cingulate and prefrontal cortices, attentional deficit impulsivity with impairments of caudate nuclei, ACC, and parietal cortical structures, and with strong activity in insular cortex; reflection impulsivity with impaired frontal lobe function; and impulsive choice with increased activation in limbic and paralimbic regions in the course of the selection of immediate rewards.

This latter is again strongly predicted by RST (McNaughton and Corr, 2008). It is debatable whether the state impulsivities mentioned here are anything other than the behavioral manifestations of trait impulsivity in particular contexts. The four state impulsivities that Bickel et al. (2012a) note are probably outcomes of a general tendency to act impulsively from which they are predictable. Behavioral disinhibition is the inability to arrest a pattern of behavior once it has started; it is also evinced in acting prematurely with deleterious outcomes. Attentional deficit impulsivity is failure to concentrate, to persevere with salient stimuli. Again, the outcome is the adoption of risky behavioral modes with poor consequences. Reflection impulsivity is failure to gather sufficient information before deciding and acting; inability to get an adequate measure of the situation leads to unrewarding behaviors. Impulsive choice is a behavioral preference for a SSR over a LLR for which the individual must wait. All of these state impulsivities are actually behaviors, the outcomes of trait impulsivity. More relevant to the present discussion is *preference reversal* in which a longer-term, more advantageous goal is preferred (e.g., verbally) at the outset only to decline dramatically in relative value as the delivery of the earlier less advantageous reward becomes imminent.

### The executive system

Bickel et al. (2012a) define EFs as “behavior that is self-directed toward altering future outcomes” (p. 363; see also Barkley, 2012) and point out that EFs are consensually associated with activity in the PFC. PFC is generally recognized as implicated in the integration of motivational information and subsequent decision-making (Wantanabe, 2009), exerting a supervisory function that governs the regulation of behavior (Bickel et al., 2012a); hence, Bickel et al. (2012a) point out, its designation as a supervisory attentional system (SAS; Shallice and Cooper, 2011).

While some authors emphasize a single element of EFs such as the attentional control of behavior or working memory or inhibition, others stress groups of elements: planning, working memory, attentional shifting or valuing future events, emotional aspects of decision-making. Addiction can then be viewed as a breakdown in the operations of the EFs or as impaired response inhibition leading to the increased salience of addiction-orientated cues. Bickel et al. (2012a) concentrate on Attention, Behavioral flexibility, Planning, Working memory, Emotional activation and self regulation (EASR) which they group into three major categories: (1) *the cross-temporal organization of behavior* (CTOB) which is concerned with the awareness of the future consequences of current or contemplated behavior and therefore with planning for events that will occur later; (2) EASR which involves the processing of emotion-related information and “initiating and maintaining goal-related responding”; and (3) *metacognition* which includes social cognition and insight, empathy, and theory of mind (ToM).

*The CTOB* comprises *attention* (closely related to dorsolateral prefrontal cortex (DLPFC), *behavioral flexibility* (frontal gyrus activity; lesioning of PFC is well-known to be associated with the diminution of behavioral flexibility (Damasio, 1994; Bechara, 2011), *behavioral inhibition* (right inferior frontal cortex and insula are activated during behavioral inhibition which is also associated with reduced activity in left DLPFC, the right frontal gyrus, right medial gyrus, left cingulate, left putamen, medial temporal, and inferior parietal cortex), *planning* (in which DLPFC the VMPFC, parietal cortex, and striatum are implicated), *valuing future events* (in the case of previewing and selecting immediate rewards: limbic and paralimbic regions; in the case of long-term decisions: prefrontal regions; see McClure et al., 2004); and *working memory* (DLPFC, VMPFC, dorsal cingulate, frontal poles, medial inferior parietal cortex, frontal gyrus, medial frontal gyrus, and precentral gyrus; Bickel et al., 2012a, pp. 363–367).

*EASR* concerned with the management of emotional responses is implemented in Medial PFC, lateral PFC, ACC, OFC. *Metacognitive processes (MP)* involve recognition of one’s own motivation and that of others which is implemented in the case of *insight* or self-awareness by the insula and ACC, and in the case of *social cognition* by medial PFC, right superior temporal gyrus, left temporal parietal junction, left somatosensory cortex, right DLPFC; moreover, *impaired social cognition* follows lesions to VMPFC (Damasio, 1994; Bechara, 2005; Bickel et al., 2012a, pp. 367–368).

### Reinforcement sensitivity and personality

RST (Gray, 1982; Corr, 2008b; Smillie, 2008) includes the excitatory (impulsivity) and inhibition (executive) components of the CNBDS model but also permits us to make extensions relating to the expected behavior patterns that follow from each and the way in which individual differences can be summed up in terms of an ascription of personality types.^1^ RST proposes that the basic behavioral processes of approach and avoidance are differentially associated with reinforcement and punishment and that individuals show variations in their sensitivity to these stimuli.^2^

Approach is behavior under the control of positively reinforcing or appetitive stimuli and is mediated by neurophysiological reward circuitry that the theory categorizes as a Behavioral Approach System or BAS. The BAS consists in the basal ganglia, especially in the mesolimbic dopaminergic system that projects from the ventral tegmental area (VTA) to the ventral striatum (notably the nucleus accumbens) and mesocortical DA PFC (Smillie, 2008; cf. Pickering and Smillie, 2008). For recent discussion of the role of the striatum in decision-making and the processing of rewards, see Delgado and Tricomi (2011). Recent research demonstrating the role of this dopaminergic system in formulating “reward prediction errors” is consonant with this understanding. Unpredicted reward is followed by increase in phasic dopaminergic activity whereas unpredicted non-reward is followed by a decrease and unchanged when reward is entirely predicted (Schultz, 2000, 2002; Schultz and Dickinson, 2000; Schultz et al., 2008). Unpredicted reward instantiates the activity of the BAS, therefore, and predicted reward maintains its operation. Moreover, BAS activity increases positive reward (pleasure) and motivates approach to reinforcing stimuli and stimuli that predict reinforcement. Such approach is characteristic of the extraverted personality; Corr (2008b, p. 10) sums up the personality type as “optimism, reward-orientation and impulsivity” and notes that it maps clinically on to addictive behaviors.

These emotional and motivational outcomes represent one pole of a continuum of individual differences that manifest differential BAS and Behavioral Inhibition System (BIS) reactions to stimuli. There is a corresponding though antithetical explanation of avoidance in RST. Avoidance is shaped by sensitivity to stimuli of punishment and threat and mediated by two bio-behaviorally based systems of emotion and motivation. The first of these, the Fight-Flight-Freeze system (FFFS), is triggered by aversive stimuli and the resulting feeling of fear, what Corr (2008b, p. 10) refers to as the “get me out of here emotion”; the FFFS’s motivational output is a behavior pattern characterized as “defensive avoidance”. However, if the consequential stimuli involved are mixed in terms of their emotional valence then the BIS, which is involved generally in the resolution of goal-conflict is activated; in this case, the emotional output is anxiety, the “watch out for danger” emotion Corr (2008b, p. 11) and the behavioral outputs are risk evaluation and cautiousness which are described as manifesting defensive approach. Hence, in summary, reward sensitivity leads to positive emotion and approach and a response pattern that is characterized as “extraversion” via behavioral observation or psychometric testing; by contrast, punishment sensitivity leads to negative emotion and avoidance and a personality characterized in terms of neuroticism (Smillie, 2008).

RST also relates the FFFS and BIS to specific neurophysiological systems. In the case of the FFFS this is the periaquedital gray, which is implicated in acute or proximal threat, and the medial hypothalamus, amygdala and interia cingulate cortex, implicated in distal threats. The BIS comprises the septo-hippocampal system and the amygdala. The emotional output of the FFFS is fearfulness while that of the BIS is anxiety. In either case, the emotional outputs are negative and most forms of RST relate this to neuroticism. The value of employing explanatory constructs referring to personality types such as extraversion and neuroticism is that they summaries individual differences in reinforcement sensitivity, adding both to the interpretation of behavior and to its prediction in novel environments.

### Managerial behavior reconsidered: the influence of temporal horizon

Dysfunctional behaviors are those *dominated* by either the impulsive system or the executive system. The impulsive system evolved because it was evolutionarily-adaptive as far as inclusive fitness was concerned. Its preoccupation with short-term goals and its immediate response to opportunities ensured its contribution to survival of the individual and thereby to its biological fitness. It is closely related to the kinds of modular functioning posited by Fodor (1983) which allows rapid responses to environmental concerns. It is closely related also to the emotion-feelings associated with such response capacity, pleasure in particular but also arousal and dominance. These are the ultimate rewards of instrumentally conditioned behavior (Rolls, 2008; Foxall, 2011).

When we speak of the dysfunctional consequences of a hyperactive impulsive system in seeking to understand and explain a manager’s behavioral repertoire we are referring to hyperactivity in these emotional-reward systems which leads, for instance, to preoccupation with short-term goals at the expense of undertaking longer-term planning, the reckless taking of investment decisions promising rapid high returns and a consequent over-cautiousness, and an unwillingness to invest in future. Another manifestation is rigidity in the pursuit of a previously selected goal even though the environment has changed and flexibility is called for. We are also suggesting that it is unlikely that this impulsive-hyperactivity occurs in isolation from hypoactivity of the executive system. Hence, imbalance occurs because managers place disproportionate importance on the emotional highs resulting from activities that result in immediate or near-immediate reinforcement at the expense of the pursuit of considered action that would be under the control of the executive system. Moreover, both utilitarian reinforcement and informational reinforcement are engendered which brings about high levels of pleasure and arousal, and in a context that permits the emotion-feeling of high dominance (Kringelbach, 2010; Foxall, 2011). This is probably the strongest combinations of interacting reinforcement for the maintenance of managerial behavior. From the organization’s point of view, if this behavioral style becomes characteristic of a function, department or even of the firm as a whole, the outcome will be an overconcentration on administrative and operational activities at the expense of a strategic perspective which embraces and anticipates the opportunities and threats of the changing market-competitive environment.

However, dysfunctional behavior may also result from hypoactivity of the impulsive system and hyperactivity of the executive system (Mojzisch and Schultz-Hardt, 2007). The intellectual rewards of a preoccupation with long-term planning, obtaining and analyzing information, mulling over strategic possibilities, may lead to a lack of strategic implementation so that the short-term decisions necessary for the day-to-day operations of the firm are neglected, working capital is lacking, the firm cannot continue. The pleasures and arousal resulting from cognitive activity and the feeling of dominance that this provides can manifest in organizational sclerosis which over-values intellectual engagement with marker structures, competition and, especially, the strategic scope of the organization. From the organization’s viewpoint, if this behavioral style becomes widespread, there will be an imbalance in favor of strategic planning and decision-making at the expense of the day-to-day imperatives of the firm’s response to the tactical behavior of competitors and the vagaries of consumer choice. The executive system also evolved because it favored biological fitness. Its operation is much like that of the central cognitive function posited by Fodor (1983).

In view of the importance of avoiding a general tendency towards either kind of imbalance in the behavior of the firm, it might be argued that our unit of analysis should be the organization as a whole since it is presumably structural elements in the organization’s culture that require attention if the problem is to be overcome. This is undeniably correct but our present objective is less to overcome problems of imbalance, which are anyway the subject of innumerable management texts, and more to understand how individual managers may be prone to one or other behavioral style. The central factor involved in diagnosing either extreme at the individual level is the temporal horizon of the manager since this correlates highly with the influence of the impulsive and/or executive systems. This is best considered, however, after the way in which cognitive language is used in neuro-behavioral decision theory, which brings further understanding of the role of temporal horizon in decision-making. It also suggests a means of overcoming problems of impulsive-hyperactivity and executive-hypoactivity at the individual level which must be evaluated before an organization-level solution can be proposed and appraised.

## The cognitive dimension

### Speaking of cognition

Neuroscience and behavioral science employ extensional language, the third-personal mode which is taken as the hallmark of science (Dennett, 1969). The truth value of extensional sentences is preserved when co-designative terms are substituted for one another. The phrase, “the fourth from the sun” can be substituted for “Mars” in the sentence “That planet is Mars” without surrendering the truth value of the sentence. However, the truth value of a sentence containing intentional language, such as “believes”, “desires” or “feels”, is not maintained when co-designatives are substituted. Given the sentence, “John believes that that planet is Mars”, we are not at liberty to say, “John believes that planet is the fourth from the sun”, since John may not know that Mars is the fourth planet. Intentional sentences have another unique property: the *intensional inexistence* of their subjects. The truth-value of my saying “I am driving to Edinburgh this weekend”, an extensionally-expressed statement, is established by there being a place called Edinburgh to which I can travel. But if I say that I am seeking the golden mountain, looking for the fountain of youth or yearning for absolute truth, none of the entities named in these intentional expressions need actually exist for the truth value of the sentences to be upheld. Finally, it is not possible to translate intentional sentences into extensional ones without altering their meaning. Intentional sentences usually take the form of an “attitude” or verb such as *believes, desires* or *wants* followed by a proposition such as “that today is Tuesday” or “that eggs are too expensive”; hence, such sentences are known as “propositional attitudes” (Chisholm, 1957).

The proposed development of the CNBDS hypothesis involves more than terminological clarification. The principles just described govern not only linguistic usage but also the kinds of theories we invoke in order to explain our subject matter and care must be taken to ensure that each is confined to the level of explanation or interpretation to which it is appropriate. Cognitive terminology is intentional and belongs only at the level of the person (Bennett and Hacker, 2003).

### Levels of exposition

Dennett (1969) distinguishes the *sub-personal level* *of explanation*, that of “brains and neuronal functioning” from the *personal level of explanation*, that of “people and minds”. The sub-personal level thus entails a separate kind of scientific purview and approach to explanation: by encompassing neuronal activity it is the domain of the neuroscientist and leads to an extensional account. The personal level which is the domain of mental phenomena is that of the psychologist; it requires an intentional account. A third level of explanation is required, however, in order to cover the whole range of phenomena and sciences that deal with them in a comprehensive approach to the explanation of behavior (Foxall, 2004). This is the *super-personal level of explanation* which encompasses operancy,^3^ the respect in which the rate of behavior is contingent upon its reinforcing and punishing consequences; this is the field of extensional behavioral science.

Care is necessary to maintain the separation of these three levels since the mode of explanation which each entails is unique and cannot be combined with the others in a simple fashion. The fundamental difference in mode of explanation which must be constantly recognized is as follows. The sub- and super-personal levels, which are based on the neuro- and behavioral-sciences respectively, require the use of extensional language and explanation. Both of which are in principle amenable to experimental (“causal”) analysis, or failing this to the quasi-causal analysis made possible by statistical inference. They differ from one another in terms of the kind of stimuli and responses (independent and dependent variables) that must be taken into consideration in empirical testing of the hypotheses to which they give rise. They differ more fundamentally from the personal level of explanation, which attracts a wholly different mode of analysis, namely that of intentional psychology; the approach to explanation in this case relies on the ascription of beliefs, desires and feelings on the basis of non-causal criteria.

The proposed development of the CNBDS hypothesis involves more than terminological clarification. The principles just described govern not only linguistic usage but also the kinds of theories we invoke in order to explain our subject matter and care must be taken to ensure that each is confined to the level of explanation or interpretation to which it is appropriate. Cognitive terminology is intentional and belongs only at the level of the person (Bennett and Hacker, 2003).

The critique of the CNBDS hypothesis takes the form therefore of conceptual development. The CNBDS hypothesis is described by Bickel and colleagues in neuroscientific, cognitive and behavioral terms without regard to the domains of explanation to which each of these categories belongs. For example, although they offer what purports to be a behavioral definition of EF, they define several of its component parts in terms that are cognitive. Following Barkley (1997a,b), they define EF as “as behavior that is self- directed toward altering future outcomes” (Bickel et al., 2012a, p. 363), but they list among those of its elements which suggest “CTOB”: *attention, planning, valuing future events* and *working memory*. These clearly are or involve cognitive events. Similarly, among the elements that make up “emotional and activation self-regulation”, they list: “the processing of emotional information” and “initiating and maintaining goal-related responding”. Finally, as elements of “MP” they list: “social cognition” or “ToM” and “insight”. Bickel et al. (2012a) define impulsivity behaviorally in terms of actions prematurely performed that eventuate in disadvantageous outcomes. They go on, however, to describe impulsivity as consisting in the trait of impulsiveness, a structural personality variable that incorporates sensation-seeking, deficits in attention and reflection impulsivity which is an inability to collect and evaluate information prior to taking a decision. All of these are intensional.

### Cognitive requirements of neuro-behavioral decision systems

So far we have advocated that behavioral and neuroscientists maintain the appropriate syntax in speaking of intentional concepts such as beliefs and desires as opposed to extensional objects such as neurons and behavior patterns. This means understanding and maintaining the sub-personal, personal and super-personal levels of exposition and employing only the appropriate language at each level. A more satisfying outcome for neuro-behavioral decision theory would be to incorporate a level of cognitive exposition the content of which complemented the extensional sciences we have discussed. This section sets out the criteria that such an account should fulfill; the following section evaluates picoeconomics (Ainslie, 1992) as that cognitive component.

There are four requirements of any candidate for the cognitive component of neuro-behavioral decision theory. It must first be capable of filling the need for a personal level account of the causes of behavior. Second, it must provide an intentional explanation. Third, it should be capable of linking to the behavioral economics and neuroeconomics analyses that are found in the hypothesis. And, finally, it must relate philosophically to broader disciplinary concerns including neurophysiology and operancy.

#### A personal level theory

A cognitive account is required to provide understanding of the ways in which individuals subjectively respond to the circumstances which influence their behavior towards rewards that may have short-term benefits but which entail longer-term deleterious consequences. Being able to characterize what individuals desire and believe in these situations, what they perceive and how they feel, provides an indication of their underlying disposition to respond in a particular way to rewards and punishments occurring at different times. This is of course a highly theoretical enterprise; in order to avoid undue speculation and conjecture, therefore, it is important that the cognitive requirements of neuro-behavioral decision theory are provided by a coherent body of knowledge relating personal level factors to situations that promote consumption.

#### An intentional account

The required personal level exposition must indicate the particular intentional terms that are applicable to the explanation of normal and addictive behaviors within the framework of an overall theory that can systematically relate the two antipodal behavior patterns. It must also be capable of explaining how intentional entities like beliefs and desires, perceptions and emotions would act upon the impulsion towards fulfillment of immediate wants, such as consumption of an addictive substance, in order to bring about a more advantageous long-term result. This calls for a well-worked out theory of human behavior over the continuum of normal to addictive behaviors rather than an ad hoc application of intentional language on the basis of rapid observation of an individual’s behavior.

#### An integrative economic account

The CNBDS hypothesis relies heavily on operant behavioral economics and neuroeconomics in order to explain the reinforcer pathologies that underlie addictive patterns of behavior. It would be advantageous, therefore, for the cognitive component of the model to link to the basic exposition in economic terms. The usefulness of the cognitive account might be questioned because of its inherently theoretical nature; this objection can be overcome if its explanation of behavior can be specified in language that is consonant with the provisions of consumption in the face of extremely high elasticity of demand and temporal discounting of the consequences of behavior.

#### Relationship to basic disciplines

A broader relationship between the cognitive account of behavior and the underlying neuroscience and behavioral science that comprise the CNBDS hypothesis is necessary that goes beyond economic integration. Although a major point of the present argument is that cognitive accounts differ fundamentally from those provided by the extensional sciences, the intentional component must be consistent with what is known of the neurophysiological basis of addiction and also with its relationship to the reinforcers and punishers that follow behavior.

### Picoeconomics: preference reversal and intertemporal conflict

Herrnstein’s (1997) matching law suggests that the value of a reinforcer is inversely proportional to its delay, i.e., as the delay becomes shorter, the value increases dramatically. This is the essence of hyperbolic discounting. The key difference between exponential and hyperbolic discounting is that in the former the LLR is always preferable to the SSR, regardless of time elapsed, whereas in the latter there is a period during which the SSR is so highly valued (because the time remaining to its possible realization is so short) that it is preferred to the LLR (Ainslie, 1992; Ainslie and Monterosso, 2003). This is clearly not because of its objective value which is by definition less than that which can be obtained through patience, but because the time remaining to its possible realization is now so short, that it is preferred to the later but larger reward. Ainslie notes that these findings harmonize with Freud’s observations that an infant behaves as if expecting immediate gratification but becomes, with experience, willing to wait for the longer-term alternative. In other words, still paraphrasing Freud, if the pleasure principle is resisted, the outcome will be the exercise of the reality principle. In the terminology of behavioral psychology, the operants relevant to each of these principles are shaped by their respective outcomes. Ainslie argues that the two principles can be represented as two *interests*, each of which seems to employ devices that undermine the other.

In discussing what these devices are, Ainslie (1992) gives a clue as to how we may speak of the operations of mental mechanisms and also how they are organized to produce phenomena in a cognitive account, i.e., one that conforms to the use of cognitive logic as we have defined it and to the strictures of grounded modularity as they were developed above. His first device, for instance, is *precommitment*, in which for instance one joins a slimming club in order to be able to call upon social pressures in order to reach long term goals. The very language of this account indicates the relevance of the models of cognition we have developed. The processes are unobservable, adopted in order to make behavior intelligible once the extensional accounts of behavioral and neuro-science have been exhausted. Secondly, the interests may hide information from one another, e.g., about the imminence of rewards. Thirdly, the emotions that control short-term responding may be incapable of suppression once they are in train or they may be foreshortened by long-term interests. Finally, current choices may be used as predictors of the whole pattern of behavior, consisting in a sequence of multiple behaviors belonging to the same operant class, that the individual will engage in future. An individual may, that is, see her present choice of a chocolate éclair as indicative that she will make this selection repeatedly and often in the future. Individual choices are thus perceived as precedents. The resulting strategy is what Ainslie later described as *bundling*, in which the outcomes of a series of future events are seen cumulatively as giving rise to a single value. When this value, rather than that of a single future event, is brought into collision with the value of the single immediate choice, the long-term interest is thereby strengthened (see also Baumeister and Vohs, 2003).

Subsequent behavior that serves the longer rather than the shorter term interest is apparently rule governed rather than contingency shaped (Skinner, 1969). However, the “rules” exist only in the mind of the individual who may not have encountered the contingencies. It is intellectually dishonest to refer to them as rules in the sense proffered by radical behaviorists which require empirical confirmation that the individual has previously encountered similar contingencies or whose rule following behavior from others of similar kind to the present has been reinforced. Since we have no empirical, in particular, experimental indication of this nature, we would more accurately refer to them as beliefs. Our use of intentional language indicates the nature of our explanation or, better perhaps, interpretation. Ainslie himself refers to bundling as the basis of “personal rules” but we can have no this- personal evidence of even the existence of such, let alone their efficacy. Better to characterize our account as interpretation and make this explicit by using intentional language.

In sum, Ainslie’s picoeconomics portrays the conflict between a smaller reward that is available sooner and a larger reward available later in terms of clashing intrapersonal interests. We can now proceed to evaluate picoeconomics in terms of the criteria set out above.

### Picoeconomics as the required cognitive component

#### A personal level account

Ainslie’s picoeconomics portrays the conflict between a smaller reward that is available sooner and a larger reward available later in terms of clashing intrapersonal interests. These are personal level events because their purpose is to render intelligible the behavior of an individual when it is no longer obvious how the contingencies of reinforcement/punishment and his neurophysiology are affecting his behavior. The behavior we are attempting to understand is often a single instance of activity (we are taking a molecular perspective) but the behavior which we employ to generate and justify the intentional interpretation we have to make is a *pattern of behavior*: here we are taking a molar standpoint. There must also be a pattern of neurophysiological activity which supports the strategic assumptions we are making about the individual. In addition, the *pattern of reinforcement* (Foxall, 2013) is of crucial importance in interpreting his behavior. We are ascribing *interests* and their effects in determining behavior but we employ constructs in order to accomplish this that are unobservable posits: they cannot enter into an experimental analysis. We use the molar behavior pattern, the pattern of reinforcement and neurophysiology to underpin these strategic assumptions and to justify our interpretation. The language of picoeconomics consists therefore in strategic assumptions that derive from an interpretation of the behavior and neurophysiology of the individual. The strategic assumptions we make and the way we use them must be consistent with the evolution of the species by natural selection, the ontogenetic development of the individual’s behavior through operancy, and the evolutionary psychology of the prevalent behavior of the species. We need to show how the behavioral sensitivity to patterns of reinforcement (which are the subject of our studies of operancy and evolutionary psychology) are in turn related to evolution by natural selection via synaptic plasticity.

#### An intentional exposition

Picoeconomics accounts for behavior using intentional language, specifically the cognitive language of decision-making and problem-solving. In particular, as a theory of “the strategic interaction of successive motivational states within the person” (Ainslie, 1992), it is dynamically concerned with the internal weighing of information about the outcomes of alternative courses of action and the motivational states they engender.

#### An economic account

Can the actions of the interests themselves be economically modeled at the intentional level? Is Ainslie’s picoeconomics entirely a cognitive theory or does it lend itself to microeconomic analysis? In fact, Ross (2012) puts forward an array of economic models of the strategic interactions proposed by picoeconomics among competing preferences. Analysis of behavior in terms of the pattern of reinforcement it has previously resulted in draws upon *operant behavioral economics* which is central to the CNBDS: specifically, the analysis of discounting relates behavior to its consequences, but operant behavioral economics also establishes that individuals maximize utility and the particular combinations of reinforcement that constitute utility.

#### Related to a broader disciplinary base

It is particularly important from the point of view of the research program within which the current investigation is being performed (see Foxall, 2007a) that the cognitive interpretation of behavior, here picoeconomics, can be defended philosophically in terms of the underlying behavioral and neuroscience (Foxall, 2004). This is clearly the case with picoeconomics (Foxall, 2007b).

Now that picoeconomics has been established as a cognitive component for neuro-behavioral decision theory, its usefulness as a means of overcoming managerial dysfunction with respect to temporal horizon can be evaluated. As Section Organization-Level Strategies for Changing Managerial Behavior indicates, the general thrust of picoeconomics is towards clinical application that may not fit most managerial situations. In that case, alternative approaches to management are discussed, notably adaption-innovation theory, which are founded on similar neurophysiological bases but which suggest more practicable solutions.

## Organization-level strategies for changing managerial behavior

### Strategies of change based on picoeconomics

An advantage of picoeconomics in the current context is that it suggests means of overcoming the managerial problems likely to arise when individual managers are strongly motivated by the goals and behavioral patterns that reflect hyperactivity in the impulsive system and hypoactivity in the executive system. Ainslie (1992) proposes a number of strategies through which the individual might overcome the temporal discounting that is the hallmark of this tendency. It is here that RST underpins the current analysis by providing neurophysiological systems that underlie not only the more extreme impulsive—approach tendency (BAS) the fear—engendered escape—avoidance tendency (FFFS), but the goal-resolving tendency that seeks to reconcile the alternative courses of action (BIS). The strategies of self-control suggested by Ainslie can be seen as attempts to aid the BIS in its attempts at conflict-resolution.

Ainslie (1992) proposes four personal strategies, allusion to some of which was made above, by which the individual might make a larger, albeit longer-term, outcome more probable: precommitment, control of attention, preparation of emotion and reward bundling. *Precommitment* involves using external commitments to preclude the irrational choice. The individual seeks to manipulate the external environment in order to make behavior leading to the LLR more likely. Ulysses lashed himself to the mast before temptations arose. But precommitment need not be so dramatic. An addict may imbibe a substance that induces nausea when alcohol is drunk. A student might arrange for friends to take her to the library before a favorite TV program begins. *Control of attention* restricts information processing with respect to the SSR. For example, taking a route home from the office that avoids bars or fast-food restaurants; thinking about the car one can buy if you eliminate cigarette smoking. *Preparation of emotion* may take the form of inhibiting emotions that are customarily connected with the SSR or of increasing incompatible emotions. Hence, graphically recalling the health risks of over-eating, smoking or excessive alcohol consumption, thinking of the displeasure others will show, engage cognitive reasoning in order to eliminate the emotional anticipation that customarily lead to consumption.

Perhaps the principal strategy, *reward bundling* requires the individual to make personal rules about the perception of the smaller-sooner and larger-later choices available. Instead of imagining the present choice and its exciting outcomes (drinking alcohol to excess) as opposed to a single somewhat amorphous outcome of sobriety (“longer life”), reward bundling involves bring a whole sequence of larger- later rewards to oppose rewards of the immediately-available behavior. In the absence of such bundling, the individual is likely to undergo repeated preference reversals but viewing the choice as between two streams of behaviors and outcomes makes self-control more possible. Self-control results from perceiving a single choice between an aggregation of LLRs and a competing aggregation of SSRs. The sum of the LLRs is always greater than that of the SSRs. Decision making is then a matter of imaginatively bringing the LLRs forward in time to the present. The personal rules necessary to ensure this self-control take the form of private “side-bets” in which the current choice predicts future choices. The important point in viewing the reward sequences in this way is that the LLR is *at all times* superior to the SSR even when an SSR is immediately available: preference reversal is therefore not predictable. The rule is a side bet that the current choice will predict future choices. If the SSR is resisted, the bet is won: the expectation of future reward is thus enhanced and the individual’s probability of success in resisting temptation is increased. Selection of the SSR indicates that the individual has lost the bet, however: the individual’s self-image is weakened, along with his or her expectation of resisting the temptation in the future.

The relevance of these strategies to managerial decision-making of the kind we have been discussing is evident though it is unclear whether a manager would be able to recognize and change his or her behavior in the absence of detailed one-on-one counseling. While this methodology obviously has applications in therapeutic contexts, and Ainslie’s prescriptions fit well the needs of substance and behavioral addicts, an application that is more attuned to the social-structural demands of organizational management is called for in the context with which we are here concerned.

### Adaption-innovation theory

There exists an alternative approach to managerial application of the neuropsychological work that has been reviewed in this paper, though the following comments are indicative and call for a dedicated research program. Adaption-innovation theory (Kirton, 2003) suggests a means of structuring decision-making groups that reflects competing neuro-behavioral systems and so avoids reliance on an individual-level prescription for managerial behavior. “Cognitive style” refers to a person’s persistent preferred manner of making decisions, the characteristic way in which they approach problems, information gathering and processing, and the kinds of solution they are likely to work towards and attempt to implement. As such, it is orthogonal to cognitive level, that is intelligence or capacity. Kirton (2003) proposes that individuals’ cognitive styles can be arrayed on a continuum from those that predispose “doing better” (the adaptive pole) to those that predispose “doing differently” (the innovative pole). Adaption-innovation is measured by the Kirton Adaption-Innovation Inventory (KAI) which evinces high levels of reliability and validity and scores correlate with a number of personality variables including extraversion and impulsivity. General population samples indicate that trait adaption-innovation is approximately normally distributed and general population scores, including of course those of managers, are arrayed over a limited continuum which falls within the theoretical spectrum of scores posited by adaption-innovation theory. In line with the purview of this paper, therefore, the managers of whom we speak are not extreme in their behaviors, though they some of them may exhibit scores towards the extremes of the bipolar construct of adaption-innovation. The behavior of the extreme adaptor is generally characterized by a tendency towards caution in decision-making and problem-solving, use of tried-and-tested methods, efficiency, rule-conformity and limited quantitative creativity manifesting in the generation of relatively few, workable solutions. The extreme innovator is, in contrast, more outlandish in selecting decisions, more likely to propose novel solutions to problems (many of which are impracticable), less efficient and more likely to modify or even break the rules. Although extraversion (measured, for example, by Eysenck’s E scale) emerges as more highly correlated with adaption-innovation (measured in the direction of the innovativeness pole), little is known about the underlying personality profiles of adaptive and innovative decision-makers in relation to the contingencies of reinforcement that shape and maintain their preferred behavioral styles. RST (Gray and McNaughton, 2000; McNaughton and Corr, 2004; Corr, 2008a) offers a means of investigating the personality profiles of decision-makers and the role of reward and punishment in their development and maintenance. This all suggests that a psychometric research program concerned with the integration of a number of fields could provide indicators for the prescription to the problems of extreme managerial style. The program would need to encompass the neurophysiology of cognition together with the psychometric measurement of personality dimensions that underlie cognitive style. Enough has been said to indicate that we understand these fields and their interactions sufficiently to embark on such a program. In the meantime, the following remarks are indicative of the work that needs to be undertaken.

### Recognizing individual differences in team-building

In contradistinction to innovators, adapters are typically prudent, using tried and tested methods, cautious, apparently impervious to boredom and unwilling to bend, let alone break, the rules. They seek the kind of efficiency that manifests in accomplishing known tasks more effectively. An extremely adaptive cognitive style suggests hyperactivity of the executive system coupled with hypoactivity of the impulsive system. Moreover, those aspects of the executive system that involve ToM, the observation of social conventions, meta-cognition, and some facets of behavioral flexibility might be adaptor characteristics that would confirm this categorization. The tentative conclusion is that adaptors would cope well and perform advantageously when involved in the intellectual, long-term, detailed thinking that strategic planning requires. The downside to their over-involvement in this kind of decision-making derives from the demands that strategic planning and commitment sometimes exert upon the ability to undertake “outside-the-paradigm” thinking. Such demands are likely to be, relatively, occasional but they are equally likely to arise at times of crisis in the market and competitive environments of firms and to benefit most from the kind of thinking which characterizes a more innovative cognitive style. In contradistinction to adaptors, innovators typically proliferate ideas that require the relatively radical change that can modify strategic direction, the product-market scope of the firm, and possibly diversification. At its extreme however, this cognitive style, suggests hypoactivity of the executive system, hyperactivity of the impulsive system. The impulsive system is geared to the rapid identification and evaluation of opportunities and threats, the capacity to envisage far-reaching, possibly disruptive, change which, in refocusing the entire strategic scope of the enterprise carries with it upheaval in working practices and both the working and non-working lives of managers and other employees. To the extent that these are innovator-traits, it is clear that decision groups need to be balanced by adaptors who can supply the capacity for sounder decision-making and the facilitators who can explain to innovators the rationale behind the behavior of adaptors, who are otherwise likely to be seen as too slow-moving to respond appropriately to the crisis, and to adaptors that which underpins the behavior of innovators who would otherwise be perceived as too outlandish to preserve the values of the organization. Innovators supply strengths in organizational decision-making: they are more likely to think outside the paradigm within which a problem has arisen, unconfined by the tried and tested methods currently in place, and to take risks. These are all relevant when the organization faces grave uncertainties and requires radical strategic reorganization. But innovators may be unsuited to more short-term decision-making which requires the skills of prudence and caution which are the hallmark of the adapter.

Normally, strategic thinking and planning require the adventurous outlook of the innovator, tempered by the prudence of the adapter. But, without top management vigilance and the planning of the teams that participate in decision-making, it might well attract a preponderance of extreme adapters. If this cognitive style dominates the strategic function, there is likely to be a dysfunctional emphasis on the planning of strategy at the expense of the taking of strategic decisions and the implementation of appropriate policies at the operational and administrative levels. Insofar as strategic decisions are unprogrammed, they therefore require the inputs of innovators. So a prolonged predominance of adapters in this role will lead to organizational imbalance. Normally, operational (and administrative) functions require the efficient involvement of the adapter, tempered by the more outward-looking tendency of the innovator. But, again without top management vigilance, they might attract the extreme innovator who seeks to take risks for short-term benefits. This will interfere with the strategic management of the enterprise and could jeopardize the overall operation of the firm.

### Level, style and structure

“Strategic” decisions do not necessarily arise at a managerial level that is automatically higher than that of any other kind of decision, nor do strategic decisions inherently involve the breaking of paradigms, and innovativeness. Just because strategy involves long-range planning does not preclude its occurring within a paradigm, albeit of grand scope, that is nevertheless known and generally-accepted; equally, the innovativeness of eroding boundaries between small-scale organizational systems should not be automatically diminished (Jablokow, 2005). Adaptive and innovative styles of cognition and creativity are constantly required, alongside one another, in the solving of problems. That which predominates appropriately in any given situation depends entirely on the specific context. Organizational problems arise when current strategies no longer fit the demands of the organizational environment: when markets, reflecting demand and competition, are no longer adequately served by the norms of organizational behavior (Jablokow and Kirton, 2009). Such changing circumstances have two vital components. The first is the changing environment must be perceived as involving precipitating events, i.e., the need for change by the organization’s leaders; it is adaptors rather than innovators who are more adept at detecting unforeseen developments that require managerial action. The second is the exploitation of the opportunities such external change is prompting, or the defensive action needed to avoid the threats that the environment contains; these tasks of advancing the required action are more likely taken effectively by the more innovative (Tubbs et al., 2012). This is a matter of cognitive style, not of cognitive or decision level.

This point is summarized by the “paradox of structure” (Kirton, 2003, pp. 126–134): while people require structure whatever their cognitive style, but that structure is ultimately stultifying as persons, organizations and environments exhibit dynamic behaviors. All the more reason for founding managerial teams and behavior patterns on the contributions of all cognitive styles.

### Neurophysiological basis of adaption—innovation

van der Molen (1994) notes on the basis of evolutionary logic that social animals are motivated by two counterposed tendencies: first, to find satisfaction in the company of conspecifics which requires a degree of cooperation and conformity; secondly, to compete with conspecifics for limited resources, such as food, sexual partners, and territory, on which individual survival and biological fitness rely. The personality characteristics which reflect these motivational forces are, in turn, “strongly intercorrelated” traits such as “self-will, thing-orientation, individualism and innovative creativity on the one pole and compliance, person-orientation, sociability, conformity and creative adaptiveness on the other. Individuals differ from one another as far as the balance between these polarities [is] concerned. This variation between individuals must have genetic components” (van der Molen, 1994, p. 140).

Drawing on the work of Cloninger (1986, 1987), van der Molen (1994, pp. 150–152; see also Skinner and Fox-Francoeur, 2013) makes a strong case for the evolutionary and genetic components of adaption—innovation. Cloninger’s “novelty-seeking” and “reward dependence” dimensions of personality are especially pertinent. The former is driven predominantly by the neurotransmitter DA which manifests in behavior that seeks to alleviate boredom and monotony, to deliver the sense of exhilaration and excitement that is generally termed “sensation-seeking” (Zuckerman, 1994); these individuals demonstrate a tendency to be “impulsive, quick-tempered and disorderly… quickly distracted or bored… easily provoked to prepare for flight or fight” (van der Molen, 1994, p. 151). “Reward dependent” individuals are, in contrast, highly dependent on “social reward and approval, sentiment and succour”; they are “eager to help and please others, persistent, industrious, warmly sympathetic, sentimental, and sensitive to social cues, praise and personal succour, but also able to delay gratification with the expectation of eventually being socially rewarded” (ibid). These individuals’ behavior is strongly controlled by the monoamine neuromodulator norepinephrine.

Which of these bundles of attributes manifests in behavior that marks out some individuals as leaders depends entirely on the demands of the managerial situation: retail banking, relying for the most part on the implementation of standard operating procedures, may have a natural tendency to encourage and reward those behaviors that reflect an adaptive cognitive style; pharmaceutical companies, whose technological, demand and competitive environments reflect a greater dynamism than is ordinarily the norm for retail banking, requires for a much larger part of its activities the presence of individuals whose cognitive and creative styles are predominantly innovative. Investment banking which is expected to reflect a large adaptively-creative style of operation but which attracts innovators is in danger of becoming the kind of “casino banking” that has been so deleterious to both corporate and general social welfare in the last decade. But the inability of an organization to achieve the right cognitive and creative accommodation to its environment will predictably culminate in catastrophe. For the retail bank whose leaders fail to perceive and respond appropriately to the changing international competition in high-street banking, the pharmaceutical firm that becomes over-involved in the development and marketing of drugs that are novel in the extreme, and for the investment bank that over-emphasizes innovative creativity to the point where reckless decisions are made, catastrophe is equally probable. Predominant organizational climate, adaptive or innovative, can be disastrous if either of these cognitive styles comes to predominate.

These behavioral styles are remarkably consonant the innovative and adaptive cognitive/creative styles, respectively, described by Kirton (2003). Their prevalence and likely genetic basis is borne out by their consistency with the RST described above (Corr, 2008a; see also Eysenck, 2006), though the terminology may vary. The incorporation of adaption-innovation theory into the framework of conceptualization and analysis also suggests a wider search for the neurophysiological basis of styles of creativity. But these are matters for further research.

## Summary and conclusions

Analyses of managerial behavior in neurophysiological terms raise two difficulties. The first is conceptual: such accounts conflate cognitive processes with neurophysiological events; the second relates to practical management: such accounts offer little by way of solution to the personal and organizational problems that result from behavior that is motivated by excess influence of either managers’ impulsive systems or their executive systems. This paper has sought to contribute to the resolution of the conceptual problem, by introducing a cognitive dimension, picoeconomics, into neuro-behavioral decision theory, and the adaption—innovation theory of cognitive styles to that of the practical problem by deriving prescriptions for changing managerial behavior.

The prime conclusion is that the use of neurophysiological theory and research in the conceptualization of managerial decision-making and in approaching the solution of problems that arise therein is entirely justified but needs to be qualified by practical considerations suggested by the nature of managerial work and the ways in which managerial behavior can be modified especially in the context of large-scale organizations. Prior to such activity, however, is the resolution of conceptual problems in the explanation of individual behavior on the basis of neurophysiological events. This paper has pursued a central requirement of neuro-behavioral decision theory’s use of intentional terminology to explain human behavior: the role of cognitive terminology and its implication for the shape of the overall theory. It has argued that picoeconomics provides a valuable means of inculcating a cognitive level of explanation into the theory and that one of its advantages is that it suggests solutions to hyperactivity in one or other of the impulsive and executive systems identified by the theory which is exacerbated by hypoactivity in the alternative system. The solutions proposed by picoeconomics may, however, be most suitable for remedial action in clinical settings rather than in organizational settings. The quest for solutions to managerial problems is more readily achieved through organization-level models of managerial activity that incorporate as fully as possible neurophysiological understandings of behavior that are compatible with those found in neuro-behavioral decision theory. One possibility in the present context is the application of adaption-innovation theory, dimensions of which are known to map reliably on to the neurophysiological and cognitive/personality factors that underpin impulsive and executive systems. The proposal that managerial teams be built and managed in ways that reflect these considerations suggests the most relevant applications of neuro- and behavioral science, with cognitive psychology, for the remediation of certain managerial excesses. These conclusions lead predictably to a call for further research along the lines indicated.

The advantage of this emphasis on cognitive style is that it differentiates managers on the basis of their susceptibility to hyper- or hypo-activity of either the impulsive or executive systems; and recognizing that the managerial functions with which we are concerned are populated by managers of widely differing cognitive styles should reduce our tendencies to stereotype managers on the basis of their broadly-defined functional roles (Foxall and Hackett, 1994; Foxall and Minkes, 1996). The neurophysiological foundations of adaption-innovation as presented here do not map directly on to those of RST or neuro-behavioral decision theory. But there is sufficient overlap to motivate further investigation.

## Conflict of interest statement

The author declares that the research was conducted in the absence of any commercial or financial relationships that could be construed as a potential conflict of interest.

## Footnotes

^1^There are several versions of RST. The present paper makes use of the fundamental elements of the version of the theory developed by Gray and McNaughton (2000), McNaughton and Corr (2004) and Corr (2008a).

^2^RST uses the term “reinforcement” to include both rewarding and punishing stimuli. This usage can be confusing in view of the confinement of “reinforcement” to instances in which consequential stimuli strengthen (i.e., increase) the rate at which a response is emitted and “punishment” to instances in which consequential stimuli reduce that rate, a usage common in behavior analysis in terms of which the CNBDS hypothesis is generally formulated. I have therefore tried to use “reinforcement” and “punishment” consistently in their behavior analytical definitions. However, it is not always possible to do justice to RST by adhering to this rule and on occasion I have used “reward” rather than “reinforcement” where this is clearer.

^3^This neologism refers to the effect on behavior of environmental contingencies of reinforcement and punishment. “Operancy”, which refers specifically to the process of reinforcement and punishment of behavior, avoids the theoretical notion of “conditioning” and is therefore more consistent with an extensional portrayal.
